# Dualistic Role of BARD1 in Cancer

**DOI:** 10.3390/genes8120375

**Published:** 2017-12-08

**Authors:** Flora Cimmino, Daniela Formicola, Mario Capasso

**Affiliations:** 1 Dipartimento di Medicina Molecolare e Biotecnologie Mediche, Università Degli Studi di Napoli “Federico II”, 80131 Naples, Italy; mario.capasso@unina.it; 2 CEINGE Biotecnologie Avanzate, 80131 Naples, Italy; 3 IRCCS SDN, Istituto di Ricerca Diagnostica e Nucleare, 80143 Naples, Italy; formicola@ceinge.unina.it

**Keywords:** BARD1, tumor suppressor, genetic variants, cancer predisposition

## Abstract

BRCA1 Associated RING Domain 1 (BARD1) encodes a protein which interacts with the N-terminal region of BRCA1 in vivo and in vitro. The full length (FL) BARD1 mRNA includes 11 exons and encodes a protein comprising of six domains (N-terminal RING-finger domain, three Ankyrin repeats and two C-terminal BRCT domains) with different functions. Emerging data suggest that BARD1 can have both tumor-suppressor gene and oncogene functions in tumor initiation and progression. Indeed, whereas FL BARD1 protein acts as tumor-suppressor with and without BRCA1 interactions, aberrant splice variants of BARD1 have been detected in various cancers and have been shown to play an oncogenic role. Further evidence for a dualistic role came with the identification of BARD1 as a neuroblastoma predisposition gene in our genome wide association study which has demonstrated that single nucleotide polymorphisms in BARD1 can correlate with risk or can protect against cancer based on their association with the expression of FL and splice variants of BARD1. This review is an overview of how BARD1 functions in tumorigenesis with opposite effects in various types of cancer.

## 1. Introduction 

**Figure 1:**
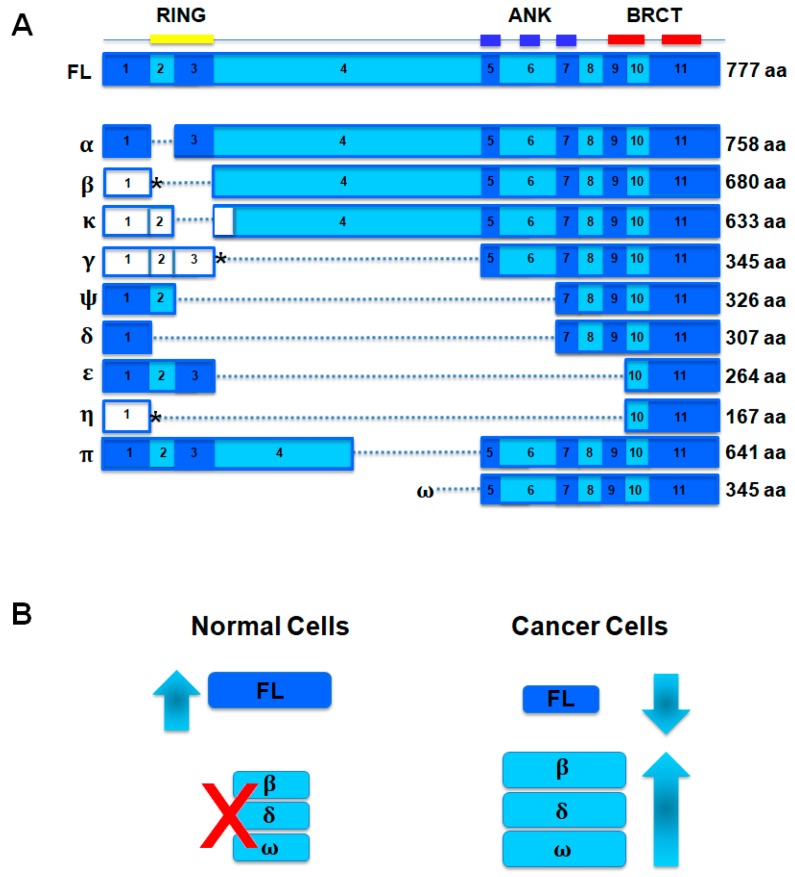
Structure of BRCA1-associated RING domain 1 (BARD1) and spliced isoforms. (**A**) Full-length (FL) BARD1 exon structure is aligned with spliced BARD1 isoforms below and protein structure above. The protein domains are reported at top of the figure. Splice variants are named with Greek letters (left). Presumed protein coding exons are shown in blue colors; non-coding exons are shown in white (β, κ, γ, η); asterisk shows alternative open reading frames (β, γ and η). Amino acid (aa) number is reported for FL BARD1 and BARD1 isoforms; (**B**) Model for dual role of BARD1 in cancer. In normal cells BARD1 isoforms (β, δ, ω isoforms, discussed in “Biological Functions of BARD1 as Oncogene” paragraph) are not expressed; in cancer cells, full-length BARD1 (FL) expression (tumor suppressor role of BARD1) decreases and BARD1 isoforms expression (oncogenic role of BARD1) increases. Figure 1 has been modified from Irmgard Irminger-Finger et al. [3].

In 1996, Wu et al. in effort to understand the function of BRCA1 they used a yeast two-hybrid screen to identify proteins that associate with it in vivo [1]. By this analysis the BRCA1-associated RING domain 1 (BARD1) protein was discovered as a binding partner of BRCA1. BARD1 protein is encoded by sequences on chromosome 2q35 and forms a functional heterodimer with BRCA1 through the binding of their RING-finger domains which functions as tumor-suppressor in breast and ovarian cancer [1,2,3,4]. The full length (FL) BARD1 mRNA includes 11 exons and encodes a protein comprising of one N-terminal RING-finger domain, three Ankyrin repeats (ANK) domains and two C-terminal BRCT domains (Figure 1). The recognizable protein motifs of BARD1 are well conserved in mouse [5,6], *Xenopus laevis* [7], *Caenorhabditis elegans* [8] and *Arabidopsis thaliana* [9], including the RING domain, the three tandem Ankyrin repeats and, to a lesser extent, the two BRCT domains. This complexity of structure indicates that BARD1 could have multiple functions.

Conditional inactivation of *Bard1* in mice induces mammary carcinomas that are indistinguishable from carcinomas induced by conditional knock-out of *Brca1*, which establishes BARD1 itself as a tumor suppressor [10]. The knock-out of *Brca1* and *Brca2* genes in mice led to embryonic lethality. Similarly, homozygous disruption of *Bard1* in mice results in lethality between embryonic days E7.5 and E8.5, at time when *Bard1* but not *Brca1* expression is maximal [5,11]. The phenotype of *Bard1* knock-out mice demonstrated that *Bard1* is essential for cell viability and maintenance of genome integrity and embryos lethality only after eight days of development could mean that *Bard1* deficiency is deleterious to the cells. This hypothesis is supported by the finding that *BARD1* mutations are associated with few cases of non-*BRCA1/BRCA2*-related sporadic breast and ovarian tumors and account for only a small fraction of cases of familial breast cancer overall [12,13,14,15,16]. Interesting to note, *BRCA1* mutations do not immediately result in malignant phenotype but have cumulative effect that is possibly caused by incorrect stoichiometry with interacting proteins [17].

The BARD1-BRCA1 heterodimer has ubiquitin ligase activity that targets proteins involved in cell-cycle regulation, DNA repair, hormone signaling and modulating chromatin structure [18,19]. Several reports show that BARD1 has an additional BRCA1-independent tumor suppressor function in cancer that is antagonized by the expression of BARD1 isoforms. Briefly, the expression of FL BARD1 (tumor suppressor role) is required for genomic stability and cell cycle control; in cancer initiation and progression the expression of BARD1 isoforms (oncogenes) antagonize FL BARD1 functions and permit uncontrolled proliferation (Figure 1B) [5,20,21,22,23]. In the following review, we have focused on the genetic and molecular mechanisms of the dualistic role of BARD1 as oncogene and tumor-suppressor in cancer.

## 2. Rare and Common Cancer-Associated Genetic Variants of *BARD1*

### 2.1. Rare Predisposing Variants of BARD1 in Cancer

Mutations in the *BRCA1* and *BRCA2* genes are the most common causes of hereditary breast and ovarian cancer and are associated with a lifetime risk of breast cancer of 50–85% and of ovarian cancer of 15–40%. It is now apparent that mutations of several other genes, such as *BARD1*, *PALB2* (Partner And Localizer Of BRCA2) and *BRIP1* (BRCA1 Interacting Protein C-Terminal Helicase 1) [24], contribute to familial breast cancer. *BARD1* mutations are expected to account for additional cases of non-*BRCA1/2* inherited breast cancer and have been reported in non-*BRCA* mutated breast cancer families [25,26,27,28]. A recent work has suggested *BARD1* as cancer-associated gene in ovarian cancer by a case-control association analysis between 1915 patients and Exome Sequencing Project (ESP, http://varianttools.sourceforge.net/Annotation/EVS) and Exome Aggregation Consortium (ExAC, http://exac.broadinstitute.org) controls [24]. The authors report a mutation frequency for *BARD1* of 0.2% and Odd Ratio of 4.2 (95% confidence interval: 1.4–12.5). Similar results have been presented by Couch et al. from multigene panel-based clinical testing for pathogenic variants in inherited cancer genes among patients with breast cancer [29]. The case-control association analysis between 38,326 white patients with breast cancer and 26,911 ExAC controls demonstrated an association between pathogenic rare variants in *BARD1* with a moderate risk value (Odd Ratio, 2.16; 95% confidence interval: 1.31–3.63) and a mutation frequency of 0.18% [29]. Thus, most of the published data are consistent with *BARD1* involvement in breast and ovarian cancers susceptibility [12,13,14,15,16,25,26,27,28,29]. Indeed, *BARD1* is now included on clinical gene panels for testing for susceptibility to these two tumors. However, no recurrent hotspot variant has been identified so far. 

Beyond single nucleotide variants, other types of risk mutations have been found in *BARD1* such as splicing mutations and large deletion. Interestingly, Ratajska et al. identified 16 *BARD1* mutations in *BRCA1/2*-negative high-risk breast and/ovarian cancer patients from Poland [30]. Among these mutations, a splice mutation (c.1315-2A > G) resulted in exon 5 skipping and a silent change (c.1977A > G) which altered several exonic splicing enhancer motifs in exon 10 and resulted in a transcript lacking exons 2–9 [30]. In a recent study, three *BARD1* mutations were identified that alter splicing leading to skipping of exons 5, 8 and 2–9, respectively [31].

**Figure 2:**
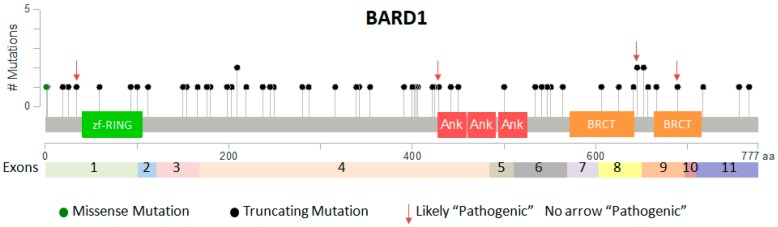
BARD1 germline coding mutations. The protein domains RING (green), Ankyrin (ANK, red), BRCA1 carboxy-terminal (BRCT, orange) are indicated. Coding mutations of BARD1 defined as “Pathogenic” and “Likely Pathogenic” in ClinVar database (https://www.ncbi.nlm.nih.gov/clinvar/) are shown. The red arrows indicate the mutations categorized as “Likely Pathogenic”, the other mutations without the arrow are categorized as “Pathogenic”.

The Table 1 shows the list of mutations (*n* = 79) defined as “Pathogenic” and “Likely Pathogenic” in ClinVar database (https://www.ncbi.nlm.nih.gov/clinvar/). Most of mutations are loss-function due to deletion, nonsense or frame shift mutations and are associated with susceptibility to breast cancer (Table 1 and Figure 2). Only one missense mutation is reported even if recent literature reports diverse potential pathogenic missense mutations in *BARD1* [24,29]. Moreover, others and we have demonstrated that *BARD1* is enriched in rare, potentially pathogenic, germline variants also in neuroblastoma patients [32,33]. Particularly, the nonsense variant (rs587781948; exon 2), included in ClinVar, has been found in two patients in these two different gene-sequence projects. Based on these observations a curated update of *BARD1* mutations in ClinVar database is needed. We also expect that massive sequencing of *BARD1* in breast, ovarian cancers, neuroblastoma and other tumors will increase the number of rare pathogenic missense mutations to be inserted into the ClinVar database as “Pathogenic”. However, these data strongly support the role of tumor-suppressor of BARD1 in different cancers.

Different copy number variants of *BARD1* locus have been found associated with congenital conditions (hypospadias and congenital heart defects: coarctation of aorta and tetralogy of fallot) and developmental phenotypes (Table 1) [34]. Neuroblastoma, tetralogy of fallot and coarctation of aorta are related to tissues that origin from neural crest cells. Moreover, literature data report cases of patients with coexisting neuroblastoma and congenital heart defects [35]. In 2004 George et al. demonstrated that congenital heart defects are more common in neuroblastoma patients than in a control group of children with another type of cancer [36]. Another study has demonstrated that depleting frog embryos of *BARD1* leads to defective developmental phenotypes (for instance: malformed neural tube and eye structures) [7]. Together, these evidences indicate that BARD1 might play a role in early organogenesis; however, additional studies are needed to demonstrate this hypothesis. 

Although variants in protein-coding regions have received the most attention, numerous studies have noted the importance of non-coding variants in cancer. A sequencing of 20 complete genes, including noncoding and flanking sequences, in hereditary breast and ovarian cancer patients (*n* = 287) identified a single nucleotide variants in 5’ UTR (c.-53G > T; rs143914387) of *BARD1* predicted to alter the mRNA structure [37]. Further complete gene sequencing or whole genome sequencing projects are warranted to investigate the contribution of rare non-coding variants of *BARD1* in conferring cancer risk. 

### 2.2. Common Predisposing Variants of BARD1 in Cancer 

Many genome-wide association studies (GWAS), using high-density single nucleotide polymorphism (SNP)-based microarray technology, have been conducted in the commonest cancer types and have identified more than 4032 genetic associations (GWAS catalog, date: 21 August 2017), confirming that susceptibility to these diseases is polygenic. We have performed a large GWAS to define the genetic landscape of sporadic neuroblastoma predisposition and have identified common DNA alleles in different genes [38,39,40,41,42,43,44,45,46,47] that are associated significantly with neuroblastoma development. In that GWAS, one of the most significant and robustly replicated association signals that was enriched in the high-risk subset of neuroblastomas resided in the *BARD1* locus [21] that is also the only neuroblastoma susceptibility gene validated in Afro-American [48], Chinese [49] and Spanish individuals [50]. We have demonstrated that, in *BARD1* locus, SNPs associated with risk of neuroblastoma correlates with high expression of splice variants of BARD1 and SNPs protecting against neuroblastoma correlates with high expression of FL BARD1 [21]. Interestingly, one disease-associated variant (rs6435862) correlates with the expression of an oncogenetically activated isoform, BARD1β, which has growth-promoting effects in neuroblastoma models potentially through cooperation with the Aurora family of kinases [51]. Furthermore, by performing a fine mapping analysis of *BARD1* locus, we have identified additionally functional polymorphisms associated with risk of neuroblastoma and over-expression of FL BARD1 [50]. These data strongly suggest that the dual role of BARD1 as oncogene or tumor-suppressor is due to the function of disease-associated variants. Together, these evidences highlight that the risk of neuroblastoma development may be estimated by a specific combination of *BARD1* risk genotypes as suggested by the results of a published computational analysis of GWAS-identified neuroblastoma risk loci [52]. 

Recently, the SNP rs7585356 previously associated to neuroblastoma has been found also associated to nephroblastoma [53], which is the most frequent malignant renal tumor in children. Although the SNP rs7585356 located in 3’ UTR of *BARD1* may have a role in BARD1 mRNA regulation, additional investigations are needed to validate this genetic association.

Candidate gene association studies have suggested that the low-frequency variant Cys557Ser (rs28997576) confers risk of single and multiple primary breast cancers in Icelandic [26] and South American [54] populations. However, independent studies failed to replicate that genetic association in Polish [55], multiethnic [56], Chinese [57], Australian [58] individuals. We also failed to validate that genetic association in a case series consisting of 540 high-risk neuroblastoma cases and 1142 controls [46] with European-American origins. These discordant results might be due to population substructure or gene modifiers affecting the role of BARD1 in cancer development. 

### 2.3. Somatic Mutations of BARD1 in Cancer

Whereas common and rare hereditable variants of *BARD1* have been associated with cancer risk, recent high-throughput sequencing studies have found no frequently acquired somatic mutations in tumor tissues. In accord to previous studies, our exome and deep sequencing of 82 clinically aggressive neuroblastomas detected only one somatic acquired mutation [32]. Interestingly, a large whole exome sequencing study on 500 metastatic cancers identified *BARD1* among the genes somatically altered at low-frequency [59] and recently *BARD1* has been included in the list of Cancer Gene Census in COSMIC database (http://cancer.sanger.ac.uk/census.). Here we have analyzed all somatic mutations of *BARD1* deposited in COSMIC database by using the Cancer-specific High-throughput Annotation of Somatic Mutations (CHASM) [60] tool to distinguish passenger variation events from driver ones across a cohort of tumors and the Variant Effect Scoring Tool (VEST) [61] to identify variants that affect the molecular function of the protein and prioritize them on the basis of the likelihood of their involvement in human disease (Table 2). We confirm that even if pathogenic somatic mutations are relatively infrequent, *BARD1* can be considered a cancer driver gene (CHASM gene score = 0.73; CHASM gene *p*-value = 0.0000004).

## 3. Biological Functions of BARD1 as Tumor Suppressor 

**Figure 3:**
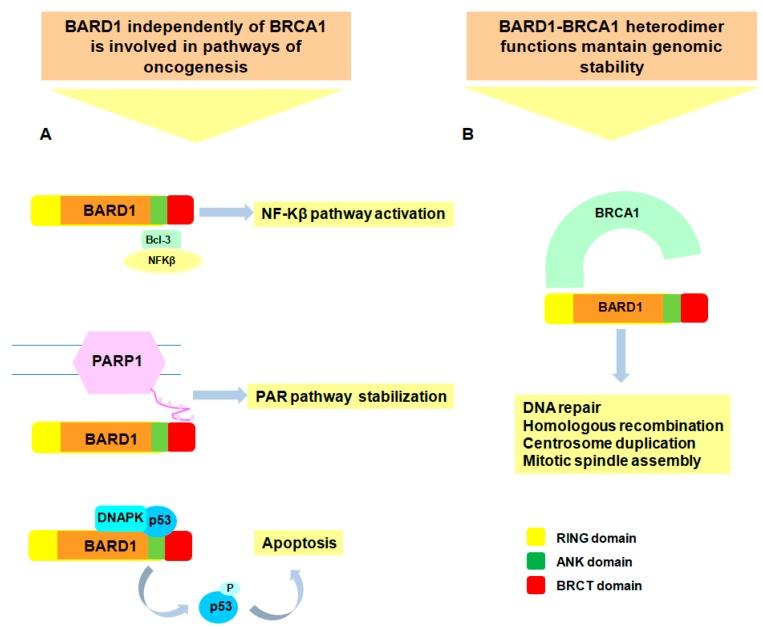
Full-length (FL) BARD1 pathways and functions. FL BARD1 participates in two major pathways as tumor suppressor. (**A**) BRCA1-independent pathways are mediated by the interaction of BARD1 with proteins involved in oncogenic pathways. BARD1 has transcriptional activity as it can induce the transcription activity of NF-κBs through binding to the NF- κB co-factor BCL3 [68]. FL BARD1 interacts to poly(ADP-ribose) (PAR) after damage and consequently it is recruited to DNA repair [69]. Finally, increased expression levels of FL BARD1 stabilize p53 and facilitate its phosphorylation by DNA-dependent protein kinase (DNAPK) [70,71,72]; (**B**) BRCA1-dependent pathways are mediated by BARD1-BRCA1 heterodimer. The activity of the BARD1-BRCA1 ubiquitin ligase is implicated in essential functions for maintaining genomic stability [61,62,63,64].

Tumor suppressor functions of BRCA1 are thought to be mediated by the BARD1-BRCA1 heterodimer which is an E3 ubiquitin ligase implicated in DNA repair [18,19] and in other essential functions for maintaining genomic stability [62,63], as homologous recombination [64], centrosome duplication [62] and mitotic spindle assembly [65] (Figure 3). Specific functions of BARD1-BRCA1 heterodimer will not be dealt with in this paragraph. Although partner of this complex, FL BARD1 initiates or facilitates DNA repair pathways by controlling polyadenylation machinery in BRCA1-independent way through BARD1 binding with mRNA polyadenylation factor cleavage stimulation factor (CSTF1) [66,67]. 

BARD1 expression fluctuates in a cell-cycle dependent manner, with maximal expression levels occurring in mitosis [73]. In mitosis FL BARD1 stability is increased due to phosphorylation by cell-cycle dependent kinase complexes (cyclin A/E-CDK2 and cyclin B-CDK2) within regions required for ubiquitin ligase activity of BARD1-BRCA1 heterodimer [74]. Contrary, BRCA1 is mostly expressed during S-phase of cell-cycle [73]. We can speculate that the concomitant expression of BARD1 and BRCA1 in S-phase support the function of BARD1-BRCA1 heterodimer and FL BARD1 expression in mitosis supports additional BRCA1-independent functions. 

BRCA1 and BARD1 have specific individual functions due to their interaction with various proteins and the dissociation of heterodimer might be regulated by post-translation protein modifications such as phosphorylation, ubiquitination or PARylation. Cancer-associated BRCA1-independent activities of BARD1 have been reported in various tumor cell lines (Figure 3). An access of monomer BARD1 over BRCA1 has been associated with *BRCA1* mutations and with p53-mediated apoptosis. The link between BARD1 and apoptosis has been further highlighted by BARD1 co-immunoprecipitation with p53 in tissues exposed to genotoxic stress [70,71]. Particularly, the region of BARD1 binding with p53 involves ANK repeats and the region between ANK and BRCT domains in BARD1-C terminal fragment [72]. It is interesting to note that mutations or deletions in *TP53* gene are frequent in cancer with *BRCA1* mutations [75,76]. We can speculate that *BRCA1* mutated tumors save BARD1 pro-apoptotic functions and additional *TP53* mutations may enhance cancer development. Contrary deleterious *BARD1* mutations are infrequent in cancer because the cells lose both DNA repair capabilities and pro-apoptotic function. BARD1 is also transcriptionally up-regulated in response to genotoxic stress and in brain after hypoxia suggesting that BARD1 is expressed specifically in tissues undergoing apoptosis [71]. 

BARD1 is involved in transcription factor NF-κB pathway. The binding of C-terminal fragment of BARD1 to the ANK repeats domain of BCL3, a NF-κB inhibitor in vitro, may affect the correct regulation of NF-κB in cancer and inflammatory and autoimmune diseases [68]. Emerging evidences report the interaction of BARD1 BRCT domain to poly(ADP-ribose) (PAR) and consequent recruitment of BARD1-BRCA1 complex to DNA repair after damage [69]. PAR pathway is particularly interesting because of the promising drugs act on inhibiting PAR polymerizing enzyme (PARP) are more efficient in cells *BRCA1* mutated with saved BARD1 tumor suppressor function. Finally, a significant association found between over-expression of FL BARD1 and favorable outcome in colon cancer patients highlighted FL BARD1 function as prognostic factor in cancer [20].

## 4. Biological Functions of BARD1 as Oncogene 

BARD1 is characterized by full length and diverse spliced isoforms (Figure 1). Down-regulation of FL BARD1 can have oncogenic effects [5,11,20,21] whereas BARD1 isoforms that lack RING or/and ANK domains are often up-regulated and associated with negative prognosis in breast [15], ovarian [15] endometrial [77] and lung [78] cancers. Several scientific evidences show that cancer-associated BARD1 isoforms antagonize the functions of FL BARD1 as tumor suppressor and act as a driving force for carcinogenesis.

BARD1β and BARD1δ isoforms were first identified in rat spermatocytes and in a highly tumorigenic and resistant to apoptosis rat ovarian cancer cell line NuTu-19 [70,79]. BARD1β is characterized by lack of exons 2 and 3 and encode to a protein lacking the RING finger and BRCA1 domain interaction. In breast and ovarian cancer an imbalance of FL BARD1 and BARD1β was observed with BARD1β dominant negative function. BARD1β scaffolds Aurora B and BRCA2 at the midbody during telophase and cytokinesis, antagonizing Aurora B ubiquitination and degradation by BARD1-BRCA1 E3 ubiquitin ligase [22]. BARD1β oncogenic driver of tumorigenesis is also supported by GWAS that identified *BARD1* as new susceptibility locus in neuroblastoma as mentioned above [51]. BARD1β depletion in vitro caused genotype-specific inhibition of cell proliferation in neuroblastoma cells, whereas overexpression of BARD1β led to the transformation of non-malignant murine fibroblast [51,77].

BARD1δ is characterized by deletion of exons 2–6 that encode for the majority of the RING finger and the entirety of the ANK repeats, critical regions for the interaction with BRCA1 and p53; this isoform was detected in many gynecological cancers and in multiple processes of tumorigenesis [70,80,81]. In MCF-7 cells, BARD1δ does not stimulate apoptosis due to p53 deficiency [80]; however, its mitochondrial localization suggested a function in regulation of mitochondrial response to tumorigenic stress [82]. Interestingly, BARD1δ specifically binds to estrogen receptor alpha (ERα) antagonizing ERα-BARD1 binding and ERα degradation [83]. To note, BARD1δ dominant negative of FL BARD1 is temporally and spatially regulated by estrogen signaling in human invasive cytotrophoblasts cells of early pregnancy [81]. Recently, Maxim Pilyugin et al. described BARD1δ antagonizes chromosome and telomere protection function of BARD1-BRCA1 heterodimer by binding molecules that confer chromosome integrity [84]. It is likely that BARD1δ confers genomic instability and acquired oncogenic property in absence of cell cycle control, due to p53 deficiency and of chromosome integrity.

BARD1ω isoform contains only exons 6–11 encoding ANK repeats and BRCT domain. This isoform was found highly expressed in acute myeloid leukemia (AML) and in AML cell lines. In vitro BARD1ω overexpression induced multiple mitotic defects like aberrant chromosome alignment at the metaphase and anaphase state, abnormally increased size of nucleus and apoptosis inhibition. These scientific evidences highlight oncogenic proprieties of BARD1ω [85].

## 5. Summary and Future Perspectives

In this review, we summarized the genetic and molecular mechanisms associated to a dualistic role of BARD1 in cancer initiation: tumor suppressor and oncogene. *BARD1* shows relatively low frequent mutations in cancer and, even if rare, *BARD1* mutations seem to drive malignant transformation. The reduced expression of FL BARD1 due to somatic mutations or predisposition gene silencing variants may be considered the first hit of BARD1 tumor suppressor function. Instead, FL BARD1 loss-of function consequently to aberrant splicing and gain of dominant negative functions is associated with its proto-oncogenic role. Indeed, cancer associated BARD1 isoforms antagonize the functions of FL BARD1 as tumor suppressor and lead to genetic instability, loss of DNA repair and cell cycle control functions and permits uncontrolled proliferation. This antagonist effect is also supported from a more recently published research article that suggests that specific microRNAs, in healthy tissues, maintain an equilibrium of FL BARD1 and isoforms in favor of FL BARD1 instead, in cancer cells, create a disequilibrium in favor of BARD1 isoforms upon epigenetic activation of non-coding BARD1 isoform BARD1 9’L [23]. 

In ClinVar database, beyond deletion, nonsense or frame shift mutations, only one missense mutation of *BARD1* is reported as “Pathogenic” even if recent literature demonstrates the association of common and rare point mutations with cancer initiation [29,86]. Thus, further functional investigations of non-coding and coding disease-associated variants are needed in order to verify their role in tumorigenesis and drug response.

BARD1 might also play a role in early organogenesis and in diseases related to tissues that origin from neural crest cells. In light of these evidences additional studies to explore BARD1 function in cancer and in developmental disorders should be considered in the next future.

### BARD1 as Possible Biomarker and Therapeutic Possibilities

BARD1β has been identified as an oncogenic driver of high-risk neuroblastoma tumorigenesis and a stabilizer of Aurora family of kinases. This strongly supports the development of potential therapeutic strategy with Aurora kinase inhibitors for clinically aggressive neuroblastoma. Moreover, the switching from FL BARD1 to BARD1β permits the deregulated turnover of the Aurora kinases. Thus, Aurora and BARD1β expression levels might be predictive biomarkers for response to Aurora inhibitors.

Protein PARylation functions as a signal to recruit DNA damage repair proteins like the BARD1-BRCA1 complex to repair Double Strand Breaks (DSBs). BARD1 BRCTs bind ADP-ribose, the basic unit of PAR, at DNA damage sites which mediates the rapid recruitment of BRCA1. PARP inhibition directly suppresses the fast recruitment of the BARD1-BRCA1 heterodimer to DNA damage sites and impairs DNA repair. PARP inhibitors (PARPi) selectively kill BRCA1-deficient cells and several PARPi are currently in breast cancer clinical trials. However, the mechanism underlying the sensitivity of the tumor cells bearing *BRCA1* mutations that abolish the interaction between BRCA1 and BARD1 to PARPi is not clear [87]. Ovarian and breast cancer patients who harbor *BRCA1* mutations develop resistance to both PARPi and platinum therapy [88,89]. Secondary mutations in *BRCA* genes as well as gene methylation status for *BRCA1*, *BRCA2* and other genes that control homologous recombination have been examined in patients’ biopsies as potential resistance mechanisms. One way to overcome clinical resistance is to investigate as the expression of FL or isoform BARD1 could contribute to the success or failure of PARPi therapy. A recent paper has demonstrated that BARD1β sensitizes colon cancer cells to poly PARP-1 inhibition even in a FL BARD1 background, thus suggesting that BARD1β may serve as a future biomarker to assess suitability of colon cancers for homologous recombination targeting with PARPi in treatment of advanced colon cancer [90]. In the future, it will be interesting to evaluate the efficacy of PARPi in patients with loss-of-function mutations of *BARD1* that are relatively frequent (Table 1).

The early detection of cancer is the most important factor contributing to the total eradication of cancer. The over-expression of BARD1 isoforms is strongly correlated with tumor progression, specifically in non-small-cell lung cancer (NSCLC) [20,51,78]. Based on these evidences Irminger-Finger et al. have developed a blood test for the early detection and diagnosis of lung cancer based on capturing autoimmune antibodies against BARD1 antigens [91]. Additional studies are needed to verify the efficacy of this test in detection of lung cancer and it will be very interesting to extend this experimentation to other cancers such as neuroblastoma, ovarian and breast cancer. 

## Figures and Tables

**Table 1:** Pathogenic and Likely pathogenic rare mutations of *BARD1* reported in ClinVar database.

Variation ID	GRCh37 Location	Ref.	Alt	Mutation Type	Protein Change	dbSNP	Frequency in ExAC Database	Condition(s)	Clinical Significance (Last Reviewed)
237823	.	.	.	deletion	.	.	.	Familial cancer of breast	Pathogenic (Last reviewed: 10 December 2015)
230523	215593433–215593434	TG	-	frameshift deletion	V767fs	rs750413473	0.00006	Familial cancer of breast|not specified|Hereditary cancer-predisposing syndrome	Conflicting interpretations of pathogenicity (Last reviewed: 18 August 2016)
185366	215593466	G	A	nonsense	W756 *	rs786202118	.	Hereditary cancer-predisposing syndrome	Pathogenic (Last reviewed: 20 June 2014)
187542	215593585–215593586	CA	-	frameshift deletion	I717fs	rs786203811	.	Hereditary cancer-predisposing syndrome	Pathogenic (Last reviewed: 10 December 2014)
422826	215593671	A	AA	frameshift duplication	D689fs	.	.	not provided	Likely pathogenic (Last reviewed: 29 November 2016)
265365	215593734	A	C	splice acceptor	.	rs876658260	.	not provided	Likely pathogenic (Last reviewed: 28 June 2016)
229902	215593734	A	T	splice acceptor	.	rs876658260	.	Hereditary cancer-predisposing syndrome	Likely pathogenic (Last reviewed: 17 November 2015)
182051	215595140	C	T	nonsense	Q666 *	rs730881422	.	Familial cancer of breast|not provided|Hereditary cancer-predisposing syndrome	Pathogenic/Likely pathogenic (Last reviewed: 13 January 2017)
187445	215595166	C	-	frameshift deletion	P657fs	rs786203739	.	Hereditary cancer-predisposing syndrome	Pathogenic (Last reviewed: 3 March 2016)
430952	215595182–215595201			frameshift duplication	Q652fs	.	.	Familial cancer of breast	Pathogenic (Last reviewed: 13 April 2017)
265510	215595182–215595201			frameshift deletion	C645fs	rs886039589	.	not provided	Likely pathogenic (Last reviewed: 3 December 2015)
127725	215595182–215595201			frameshift duplication	Q652fs	rs587780024	.	Familial cancer of breast|not provided|Hereditary cancer-predisposing syndrome	Pathogenic/Likely pathogenic (Last reviewed: 13 April 2017)
142499	215595203–215595204	AT	-	frameshift deletion	C645fs	rs587782504	.	Hereditary cancer-predisposing syndrome	Pathogenic/Likely pathogenic (Last reviewed: 20 November 2015)
141702	215595215	C	T	nonsense	R641 *	rs587781948	0.00001	Familial cancer of breast|not provided|Hereditary cancer-predisposing syndrome	Pathogenic (Last reviewed: 4 October 2016)
232108	215595234	A	-	splice acceptor	.	rs876659560	.	Hereditary cancer-predisposing syndrome	Likely pathogenic (Last reviewed: 29 May 2015)
219763	215595234	A	T	splice acceptor	.	rs864622239	.	Familial cancer of breast	Likely pathogenic (Last reviewed: 8 August 2015)
233167	215609790	G	T	splice donor	.	rs876660237	.	Hereditary cancer-predisposing syndrome	Likely pathogenic (Last reviewed: 6 August 2015)
232127	215609822	T	-	frameshift deletion	L625fs	rs876659572	.	not provided|Hereditary cancer-predisposing syndrome	Pathogenic (Last reviewed: 26 July 2016)
143017	215609876–215609877	AT	-	frameshift deletion	H606fs	rs587782897	.	not provided|Hereditary cancer-predisposing syndrome	Pathogenic (Last reviewed: 8 June 2016)
245801	215609884	G	A	splice acceptor	.	rs879253952	.	not provided	Pathogenic (Last reviewed: 9 June 2015)
246449	215609885–215609893		-	splice acceptor	.	rs879254264	.	not provided	Likely pathogenic (Last reviewed: 7 March 2016)
232643	215610445	G	A	splice donor	.	rs876659894	.	Hereditary cancer-predisposing syndrome	Likely pathogenic (Last reviewed: 30 June 2015)
127720	215610566	C	T	nonsense	Q564*	rs587780021	0.00005	Familial cancer of breast|not provided|Hereditary cancer-predisposing syndrome	Pathogenic (Last reviewed: 3 January 2017)
406791	215617170	G	C	splice donor	.	rs1060501310	.	Familial cancer of breast	Likely pathogenic (Last reviewed: 2 August 2016)
141384	215617196	C	G	nonsense	S551 *	rs587781707	.	Familial cancer of breast|not provided|Hereditary cancer-predisposing syndrome	Pathogenic (Last reviewed: 29 December 2016)
230172	215617209	G	T	nonsense	E547 *	rs876658429	.	Hereditary cancer-predisposing syndrome	Pathogenic (Last reviewed: 28 January 2015)
406768	215617214–215617248			frameshift indel	T534fs	rs1064792931	.	Familial cancer of breast	Pathogenic (Last reviewed: 22 September 2016)
379750	215617226	C	A	nonsense	S541 *	rs777937955	.	not provided	Pathogenic (Last reviewed: 14 May 2015)
406776	215632275	-	A	frameshift duplication	D500fs	.	.	Familial cancer of breast	Pathogenic (Last reviewed: 13 August 2016)
421820	215633955	-	G	splice donor duplication	.	.	.	not provided	Likely pathogenic (Last reviewed: 4 August 2016)
233414	215634002	-	A	frameshift duplication	N450fs	rs876660390	.	Hereditary cancer-predisposing syndrome	Pathogenic (Last reviewed: 17 August 2015)
406748	215634026	C	-	frameshift deletion	P442fs	rs1060501287	.	Familial cancer of breast|not provided	Pathogenic/Likely pathogenic (Last reviewed: 20 September 2016)
243116	215645314	A	-	frameshift deletion	E429fs	rs879253879	.	not provided	Likely pathogenic (Last reviewed: 11 December 2015)
234190	215645328	A	-	frameshift deletion	R424fs	rs876660911	.	Hereditary cancer-predisposing syndrome	Pathogenic (Last reviewed: 2 October 2015)
187182	215645331–215645334	GTG	ATG	frameshift indel	V422fs	rs786203533	.	Hereditary cancer-predisposing syndrome	Pathogenic (Last reviewed: 10 November 2014)
229677	215645382	C	T	nonsense	R406 *	rs377153250	0.00003	Hereditary cancer-predisposing syndrome	Pathogenic (Last reviewed: 11 November 2014)
142734	215645386	C	G	nonsense	Y404 *	rs587782681	.	Familial cancer of breast|not provided|Hereditary cancer-predisposing syndrome	Pathogenic (Last reviewed: 13 October 2016)
379884	215645393	C	G	nonsense	S402 *	rs796666047	.	not provided	Pathogenic (Last reviewed: 1 June 2015)
187646	215645400	A	-	frameshift deletion	S400fs	rs786203891	.	Hereditary cancer-predisposing syndrome	Pathogenic (Last reviewed: 17 December 2014)
237814	215645426	C	G	nonsense	S391 *	rs878853995	.	Familial cancer of breast	Pathogenic (Last reviewed: 24 February 2016)
185916	215645537	C	G	nonsense	S354 *	rs786202559	.	Hereditary cancer-predisposing syndrome	Pathogenic (Last reviewed: 24 March 2016)
232902	215645575	G	-	frameshift deletion	S342fs	rs876660061	.	not provided|Hereditary cancer-predisposing syndrome	Pathogenic (Last reviewed: 21 July 2015)
419156	215645584	C	-	frameshift deletion	S339fs	rs1064793682	.	not provided	Pathogenic (Last reviewed: 1 June 2015)
141412	215645651	T	G	nonsense	L316 *	rs587781728	.	Hereditary cancer-predisposing syndrome	Pathogenic (Last reviewed: 7 April 2014)
185015	215645737–215645738	AG	-	frameshift deletion	E287fs	rs786201868	.	Hereditary cancer-predisposing syndrome	Pathogenic (Last reviewed: 29 May 2014)
232418	215645759–215645760	TT	-	frameshift deletion	L280fs	rs876659752	.	Hereditary cancer-predisposing syndrome	Pathogenic (Last reviewed: 15 June 2015)
406757	215645853	-	A	frameshift duplication	I249fs	.	.	Familial cancer of breast	Pathogenic (Last reviewed: 24 October 2016)
141005	215645865	C	T	nonsense	Q245 *	rs587781430	0.00001	not provided|Hereditary cancer-predisposing syndrome	Pathogenic (Last reviewed: 8 March 2017)
230107	215645889	C	T	nonsense	Q237 *	rs587780035	.	not provided|Hereditary cancer-predisposing syndrome	Pathogenic (Last reviewed: 11 July 2016)
141342	215645938–215645941	TTTA	-	frameshift deletion	R219fs	rs587781671	.	Hereditary cancer-predisposing syndrome	Pathogenic (Last reviewed: 23 February 2014)
219726	215645970–215645971	AA	-	frameshift deletion	K209fs	rs864622223	.	Familial cancer of breast|not provided	Pathogenic/Likely pathogenic (Last reviewed: 4 December 2015)
127742	215645975	-	A	frameshift deletion	K209fs	rs587780033	.	Familial cancer of breast|Hereditary cancer-predisposing syndrome	Pathogenic (Last reviewed: 18 November 2016)
182042	215645991	G	T	nonsense	G203 *	rs730881415	.	not provided	Pathogenic (Last reviewed: 25 September 2014)
233965	215646006	G	-	frameshift deletion	A198fs	rs876660761	.	Hereditary cancer-predisposing syndrome	Pathogenic (Last reviewed: 15 September 2015)
186576	215646058–215646059	AT	-	frameshift deletion	Y180fs	rs779427628	0.00001	Familial cancer of breast|not provided|Hereditary cancer-predisposing syndrome	Pathogenic (Last reviewed: 13 December 2016)
406761	215646072	C	T	nonsense	Q176 *	rs776851287	.	Familial cancer of breast	Pathogenic (Last reviewed: 26 May 2016)
185846	215646102	C	T	nonsense	Q166 *	rs786202500	.	not provided|Hereditary cancer-predisposing syndrome	Pathogenic (Last reviewed: 4 April 2016)
230344	215646138–215646141	-	AAAG	frameshift duplication	V154fs	rs772486760	.	Hereditary cancer-predisposing syndrome	Pathogenic (Last reviewed: 2 February 2015)
182036	215646150	C	T	nonsense	R150 *	rs730881411	0.00001	Familial cancer of breast|not provided|Hereditary cancer-predisposing syndrome	Pathogenic (Last reviewed: 29 July 2016)
185139	215657051	C	T	nonsense	R112 *	rs758972589	0.00001	Familial cancer of breast|not provided|Hereditary cancer-predisposing syndrome	Pathogenic (Last reviewed: 27 May 2016)
185079	215657087	C	T	nonsense	Q100 *	rs786201912	.	Familial cancer of breast|Hereditary cancer-predisposing syndrome	Pathogenic (Last reviewed: 13 December 2016)
230447	215657108	C	T	nonsense	Q93 *	rs876658571	.	Hereditary cancer-predisposing syndrome	Pathogenic (Last reviewed: 23 February 2015)
231019	215657170	G	A	splice acceptor	.	rs876658905	.	Hereditary cancer-predisposing syndrome	Likely pathogenic (Last reviewed: 26 March 2015)
371931	215661823–215661824	AG	-	frameshift deletion	E59fs	rs1057517589	.	Familial cancer of breast	Pathogenic/Likely pathogenic (Last reviewed: 16 November 2016)
246176	215661842	G	T	splice acceptor	.	rs879254139	.	not provided	Likely pathogenic (Last reviewed: 3 December 2015)
246476	215674192	G	A	nonsense	W34 *	rs879254280	.	not provided	Likely pathogenic (Last reviewed: 8 March 2016)
231232	215674224–215674225			frameshift indel	A25fs	rs876659040	.	Hereditary cancer-predisposing syndrome	Pathogenic (Last reviewed: 25 March 2015)
233594	215674239	G	T	nonsense	E19 *	rs752514155	0.00002	Familial cancer of breast|not provided|Hereditary cancer-predisposing syndrome	Pathogenic (Last reviewed: 28 August 2016)
232926	215674271–215674290			frameshift deletion	P2fs	rs876660077	.	Hereditary cancer-predisposing syndrome	Pathogenic (Last reviewed: 10 July 2015)
127739	215674291	G	A	missense	M1I	rs587780031	.	not provided	Pathogenic (Last reviewed: 5 November 2013)
154206	190300875–242783384	.	.	copy number gain (2q32.2-37.3)	.	nssv3395386	.	Hypospadias	Pathogenic (Last reviewed: 18 March 2014)
152738	193803552–216569775	.	.	copy number loss (2q32.3-35)	.	nssv1608180	.	Developmental delay AND/OR other significant developmental or morphological phenotypes	Pathogenic (Last reviewed: 14 January 2013)
150620	181378520–225167565	.	.	copy number gain (2q31.3-36.1)	.	nssv1604018	.	Developmental delay AND/OR other significant developmental or morphological phenotypes	Pathogenic (Last reviewed: 25 July 2011)
146683	211444400–243059659	.	.	copy number gain (2q34-37.3)	.	nssv706486	.	Coarctation of aorta	Pathogenic (Last reviewed: 25 February 2011)
59164	213479146–227985946	.	.	copy number gain (2q34-36)	.	nssv578841	.	Developmental delay AND/OR other significant developmental or morphological phenotypes	Pathogenic (Last reviewed: 12 August 2011)
59160	193987039–242014395	.	.	copy number gain (2q32.3-37.3)	.	nssv578835	.	Developmental delay AND/OR other significant developmental or morphological phenotypes	Pathogenic (Last reviewed: 12 August 2011)
59159	191175462–242834921	.	.	copy number gain (2q32.2-37.3)	.	nssv578834	.	Developmental delay AND/OR other significant developmental or morphological phenotypes	Pathogenic (Last reviewed: 12 August 2011)
59158	189682921–243007359	.	.	copy number gain (2q32.2-37.3)	.	nssv578833	.	Developmental delay AND/OR other significant developmental or morphological phenotypes	Pathogenic (Last reviewed: 12 August 2011)
57419	195763507–237382556	.	.	copy number gain (2q32.3-37.3)	.	nssv578837	.	Developmental delay AND/OR other significant developmental or morphological phenotypes	Pathogenic (Last reviewed: 12 August 2011)

*: truncated protein.

**Table 2:** Somatic mutations of *BARD1* deposited in COSMIC database with pathogenicity statistical significance.

Position	Sequence Ontology	Protein Sequence Change	CHASM *p*-Value	VEST *p*-Value	ID dbSNP	Frequency in ExAC Database	COSMIC Variant Count in Tissues (*n*)
214769310	SG	R123 *		0.1290	rs369986649	0.00	large_intestine (1); lung (1); endometrium (1); skin(2)
214781072	MS	E268K	0.2724	0.4382		0.00	cervix(1); liver(2)
214728840	MS	A724T	0.0462	0.0145		0.00	large_intestine(2)
214730417	MS	E665D	0.3204	0.5076		0.00	liver(2)
214728936	MS	I692F	0.4718	0.2635		0.00	liver(2)
214781454	MS	K140N	0.5620	0.1730	rs758749603	0.000008	large_intestine(1); endometrium(1)
214781285	SG	K197 *		0.2026		0.00	liver(2)
214781251	FD	K208RKL *		0.0588		0.000017	large_intestine(1)
214780799	SY	L359L				0.00	esophagus(2)
214730452	MS	P654S	0.0970	0.6800		0.00	skin(2)
214792396	MS	P89A	0.2542	0.0534		0.00	pancreas(2)
214728907	MS	Q701H	0.1730	0.1709		0.00	esophagus(1)
214781390	MS	S162A	0.5712	0.2689		0.00	hematopoietic_and_lymphoid_tissue(2)
214780783	MS	S364L	0.4934	0.7981	rs200168806	0.00	esophagus(2)
214780784	MS	S364T	0.7164	0.3940	rs201292946	0.00	esophagus(2)
214780955	MS	Y307N	0.6760	0.5594		0.00	esophagus(1)
214769314	SS			0.0374		0.00	
214781510	SS			0.1037		0.00	
214728990	SG	G674 *		0.0577		0.00	
214745084	SG	W629 *		0.0191	rs747446711	0.000008	lung(1)
214780882	MS	P331R	0.6070	0.2104		0.00	autonomic_ganglia(1)
214780655	MS	V407M	0.2596	0.8458		0.00	biliary_tract(1)
214781361	^ FD	see footnote		0.2006		0.000016	biliary_tract(1)
214781136	SY	P246P			rs587780859	0.000096	bone(1)
214781165	MS	Q237E	0.4816	0.8826	rs587780035	0.000096	bone(1)
214745777	SY	L585L				0.00	breast(1)
214745791	MS	Q581K	0.3898	0.0927		0.00	breast(1)
214792394	SY	P89P			rs756165637	0.000008	breast(1)
214781066	MS	E270K	0.3180	0.3405		0.00	cervix(1)
214781210	MS	A222S	0.8046	0.7358		0.00	cervix(1)
214781384	SG	Q164 *		0.1407		0.00	endometrium(1)
214781306	MS	D190Y	0.6800	0.5193	rs369561166	0.000008	endometrium(1)
214781158	MS	L239R	0.4934	0.5867		0.00	endometrium(1)
214780657	MS	R406Q	0.6028	0.7262	rs587780014	0.0000412	endometrium(1)
214730490	MS	R641Q	0.2660	0.6668	rs752870879	0.0000082	endometrium(1)
214781449	SG	S142 *		0.1272		0.00	endometrium(1)
214745097	MS	L625I	0.5980	0.2316		0.00	endometrium(1)
214780981	MS	V298A	0.4174	0.9235		0.00	endometrium(1)
214728928	SY	L694L			rs139620052	0.000157	endometrium(1)
214745741	SY	Y597Y				0.00	endometrium(1)
214767501	MS	G517R	0.2520	0.0149		0.00	hematopoietic_and_lymphoid_tissue(1)
214767558	MS	L498I	0.1770	0.0629		0.00	
214730463	MS	K650T	0.3204	0.2411		0.00	kidney(1)
214767483	MS	V523I	0.2358	0.4735		0.00	kidney(1)
214767560	MS	P497L	0.0730	0.0046		0.00	kidney(1)
214781275	MS	A200V	0.3100	0.7394		0.00	kidney(1)
214792349	MS	M104I	0.1630	0.0450	rs752133770	0.000008	kidney(1)
214728757	SY	R751R			rs750001065	0.00	large_intestine(1)
214728786	MS	L742V	0.4234	0.7816		0.00	large_intestine(1)
214728839	MS	A724V	0.0356	0.0153	rs587782662	0.000041	large_intestine(1)
214728971	SG	W680 *		0.0967		0.00	large_intestine(1)
214745779	MS	L585F	0.2630	0.0431		0.00	large_intestine(1)
214752448	MS	V559A	0.0894	0.2909		0.00	large_intestine(1)
214767651	MS	E467K	0.1858	0.0168		0.00	large_intestine(1)
214780632	MS	M414I	0.7050	0.5045		0.00	large_intestine(1)
214780885	MS	S330I	0.6160	0.6216		0.00	large_intestine(1)
214780904	MS	H324N	0.6444	0.6244		0.00	large_intestine(1)
214780960	MS	K305T	0.7818	0.5088		0.00	large_intestine(1)
214780974	SY	P300P				0.00	large_intestine(1)
214781327	MS	V183L	0.5944	0.3666		0.00	large_intestine(1)
214781495	MS	K127E	0.7706	0.7394		0.00	
214792327	SG	R112 *		0.0323	rs758972589	0.000008	large_intestine(1)
214752523	MS	T534K	0.3864	0.0564		0.00	
214752535	MS	P530L	0.0214	0.0078		0.00	liver(1)
214781157	SY	L239L			rs760951875	0.000009	
214781422	MS	S151N	0.3204	0.1398		0.00	liver(1)
214792355	MS	D102E	0.5138	0.1463		0.00	
214809564	SY	P2P				0.00	liver(1)
214728989	MS	G674V	0.1270	0.0055		0.00	lung(1)
214730432	MS	S660R	0.2322	0.0874		0.00	lung(1)
214745727	MS	S602I	0.6860	0.1905		0.00	lung(1)
214745805	MS	G576V	0.4174	0.1383		0.00	lung(1)
214752450	SY	S558S				0.00	lung(1)
214767507	MS	S515A	0.5474	0.5937		0.00	lung(1)
214767582	MS	T490P	0.4354	0.5565		0.00	lung(1)
214780637	MS	A413T	0.3100	0.8492		0.00	lung(1)
214780642	MS	P411H	0.3420	0.0515		0.00	lung(1)
214780818	SY	V352V			rs768469265	0.000008	lung(1)
214780842	MS	S344R	0.6530	0.5045		0.00	lung(1)
214780856	MS	I340F	0.8810	0.8826		0.00	
214781201	MS	E225K	0.2884	0.6445		0.00	
214781353	MS	S174I	0.7774	0.7572		0.00	lung(1)
214781387	MS	V163M	0.2284	0.3354		0.00	lung(1)
214797074	MS	H68Y	0.0190	0.0139		0.00	
214809509	MS	R21C	0.2122	0.7193		0.00	lung(1)
214769252	MS	H459Y	0.2160	0.2473		0.00	NS(1)
214769253	SY	D458D				0.00	NS(1)
214769270	MS	D453N	0.2630	0.1696		0.00	NS(1)
214809491	MS	E27K	0.1308	0.5288		0.00	NS(1)
214728731	MS	S760L	0.2122	0.0823	rs730881425	0.000025	esophagus(1)
214728819	MS	R731C	0.2852	0.3666	rs76744638	0.000041	esophagus(1)
214745127	MS	Q615E	0.4234	0.1915	rs751710099	0.000008	esophagus(1)
214767505	SY	S515S				0.00	esophagus(1)
214767573	MS	Q493E	0.5370	0.5719		0.00	esophagus(1)
214780709	MS	S389R	0.5944	0.4714		0.00	esophagus(1)
214781120	MS	P252S	0.3204	0.8220	rs758368819	0.000009	esophagus(1)
214781351	MS	A175P	0.8810	0.4547		0.00	esophagus(1)
214745744	SY	K596K			rs777084777	0.000066	pancreas(1)
214752510	SY	S538S			rs781448650	0.000016	pancreas(1)
214767635	MS	G472E	0.1042	0.0035		0.00	pancreas(1)
214792385	MS	I92M	0.4354	0.2288		0.00	pancreas(1)
214780909	MS	R322H	0.6340	0.7262	rs774251286	0.000017	pituitary(1)
214730417	SY	E665E				0.00	prostate(1)
214752536	MS	P530S	0.0144	0.0102	rs760144724	0.000008	prostate(1)
214769261	MS	V456I	0.1904	0.4630		0.00	prostate(1)
214781033	MS	P281S	0.1358	0.6871	rs200059956	0.000017	prostate(1)
214781127	SY	I249I			rs746551077	0.000009	prostate(1)
214781355	SY	A173A				0.00	prostate(1)
214728723	MS	F763V	0.6444	0.3044	rs761586960	0.000008	skin(1)
214730442	MS	P657L	0.0304	0.0144		0.00	skin(1)
214730443	MS	P657S	0.1270	0.1673		0.00	
214745129	MS	V614A	0.1114	0.5492		0.00	skin(1)
214745834	SY	R566R				0.00	skin(1)
214767584	MS	T489I	0.1730	0.2288		0.00	skin(1)
214767639	MS	H471Y	0.2284	0.1872	rs867587389	0.00	skin(1)
214769288	MS	L447F	0.1204	0.0426		0.00	skin(1)
214780570	MS	A435V	0.3420	0.0168		0.00	skin(1)
214780609	MS	V422G	0.8136	0.8197	rs76824305	0.000232	skin(1)
214780643	MS	P411S	0.0962	0.1509		0.00	skin(1)
214780882	MS	P331L	0.3898	0.2518		0.00	skin(1)
214781320	MS	P185L	0.4934	0.8492		0.00	skin(1)
214781323	MS	S184F	0.1858	0.2233	rs184660818	0.00	skin(1)
214792395	MS	P89Q	0.1056	0.0249		0.00	skin(1)
214792396	MS	P89S	0.0212	0.0417		0.00	skin(1)
214809508	MS	R21L	0.2038	0.6894		0.00	skin(1)
214728752	MS	G753D	0.0762	0.0168	rs867281641	0.00	stomach(1)
214728780	MS	N744D	0.5416	0.4141		0.00	stomach(1)
214728831	MS	D727N	0.3044	0.5867	rs730881424	0.000025	stomach(1)
214728891	MS	P707S	0.0436	0.0289		0.00	stomach(1)
214728985	SY	C675C				0.00	stomach(1)
214730411	SY	L667L				0.00	stomach(1)
214767511	SY	L513L				0.00	stomach(1)
214767611	MS	L480S	0.1242	0.0086	rs149839922	0.000008	stomach(1)
214769254	MS	D458V	0.0432	0.0034		0.00	stomach(1)
214780751	MS	T375A	0.5088	0.8954		0.00	stomach(1)
214781250	FI	K208KENFS *		0.0618	rs587780033	0.000017	stomach(1)
214781418	SY	K152K				0.00	stomach(1)
214792416	MS	G82V	0.4234	0.2775		0.00	stomach(1)
214745083	MS	W629C	0.1678	0.0025		0.00	
214781258	MS	Q206K	0.4174	0.6353		0.00	
214728689	MS	P774L	0.3692	0.1073		0.00	upper_aerodigestive_tract(1)
214745730	MS	D601G	0.6122	0.1612	rs767765131	0.000016	
214769271	MS	S452R	0.5370	0.1476		0.00	upper_aerodigestive_tract(1)
214781182	MS	S231C	0.5654	0.5912		0.00	upper_aerodigestive_tract(1)
214809454	MS	A39G	0.5572	0.4215		0.00	
214745154	MS	H606D	0.1552	0.0017		0.00	urinary_tract(1)
214781269	MS	S202C	0.3982	0.4655		0.00	urinary_tract(1)

SG: Stop Gain; MS: Missense; FD: Frameshift Deletion; SY: Synonymous; SS: Splicing Site; FI: Frameshift Insertion; *: truncated proteins; ^: protein deletion K171KMQVLSKTHMNLFPQVLLQMFLRGLKRLLQDLEKSKKRKL; ID dbSNP: Nomenclature of the single nucleotide polymorphism; Exac database (http://exac.broadinstitute.org/): Exome Aggregation Consortium is a database that reports the frequency of variants from a wide variety of large-scale sequencing projects, CHASM: Cancer-specific High-throughput Annotation of Somatic Mutations; VEST: Variant Effect Scoring Tool.

## References

[1] Wu L.C., Wang Z.W., Tsan J.T., Spillman M.A., Phung A., Xu X.L., Yang M.C., Hwang L.Y., Bowcock A.M., Baer R. (1996). Identification of a RING protein that can interact *in vivo* with the *BRCA1* gene product. Nat. Genet..

[2] Brzovic P.S., Meza J.E., King M.C., Klevit R.E. (2001). BRCA1 RING domain cancer-predisposing mutations. Structural consequences and effects on protein-protein interactions. J. Biol. Chem..

[3] Irminger-Finger I., Ratajska M., Pilyugin M. (2016). New concepts on BARD1: Regulator of BRCA pathways and beyond. Int. J. Biochem. Cell Biol..

[4] Irminger-Finger I., Jefford C.E. (2006). Is there more to BARD1 than BRCA1?. Nat. Rev. Cancer.

[5] Irminger-Finger I., Soriano J.V., Vaudan G., Montesano R., Sappino A.P. (1998). In Vitro repression of Brca1-associated RING domain gene, *Bard1*, induces phenotypic changes in mammary epithelial cells. J. Cell Biol..

[6] Ayi T.C., Tsan J.T., Hwang L.Y., Bowcock A.M., Baer R. (1998). Conservation of function and primary structure in the BRCA1-associated RING domain (BARD1) protein. Oncogene.

[7] Joukov V., Chen J., Fox E.A., Green J.B., Livingston D.M. (2001). Functional communication between endogenous BRCA1 and its partner, BARD1, during *Xenopus laevis* development. Proc. Natl. Acad. Sci. USA.

[8] Boulton S.J., Martin J.S., Polanowska J., Hill D.E., Gartner A., Vidal M. (2004). BRCA1/BARD1 orthologs required for DNA repair in *Caenorhabditis elegans*. Curr. Biol..

[9] Lafarge S., Montane M.H. (2003). Characterization of *Arabidopsis thaliana* ortholog of the human breast cancer susceptibility gene 1: *AtBRCA1*, strongly induced by gamma rays. Nucleic Acids Res..

[10] Shakya R., Szabolcs M., McCarthy E., Ospina E., Basso K., Nandula S., Murty V., Baer R., Ludwig T. (2008). The basal-like mammary carcinomas induced by *Brca1* or *Bard1* inactivation implicate the BRCA1/BARD1 heterodimer in tumor suppression. Proc. Natl. Acad. Sci. USA.

[11] McCarthy E.E., Celebi J.T., Baer R., Ludwig T. (2003). Loss of Bard1, the heterodimeric partner of the Brca1 tumor suppressor, results in early embryonic lethality and chromosomal instability. Mol. Cell. Biol..

[12] Ghimenti C., Sensi E., Presciuttini S., Brunetti I.M., Conte P., Bevilacqua G., Caligo M.A. (2002). Germline mutations of the BRCA1-associated RING domain (BARD1) gene in breast and breast/ovarian families negative for BRCA1 and BRCA2 alterations. Genes Chromosomes Cancer.

[13] Ishitobi M., Miyoshi Y., Hasegawa S., Egawa C., Tamaki Y., Monden M., Noguchi S. (2003). Mutational analysis of *BARD1* in familial breast cancer patients in Japan. Cancer Lett..

[14] Karppinen S.M., Heikkinen K., Rapakko K., Winqvist R. (2004). Mutation screening of the *BARD1* gene: Evidence for involvement of the Cys557Ser allele in hereditary susceptibility to breast cancer. J. Med. Genet..

[15] Wu J.Y., Vlastos A.T., Pelte M.F., Caligo M.A., Bianco A., Krause K.H., Laurent G.J., Irminger-Finger I. (2006). Aberrant expression of *BARD1* in breast and ovarian cancers with poor prognosis. Int. J. Cancer.

[16] Irminger-Finger I. (2010). BARD1, a possible biomarker for breast and ovarian cancer. Gynecol. Oncol..

[17] Deng C.X. (2006). BRCA1: Cell cycle checkpoint, genetic instability, DNA damage response and cancer evolution. Nucleic Acids Res..

[18] Hashizume R., Fukuda M., Maeda I., Nishikawa H., Oyake D., Yabuki Y., Ogata H., Ohta T. (2001). The RING heterodimer BRCA1-BARD1 is a ubiquitin ligase inactivated by a breast cancer-derived mutation. J. Biol. Chem..

[19] Baer R., Ludwig T. (2002). The BRCA1/BARD1 heterodimer, a tumor suppressor complex with ubiquitin E3 ligase activity. Curr. Opin. Genet. Dev..

[20] Sporn J.C., Hothorn T., Jung B. (2011). BARD1 expression predicts outcome in colon cancer. Clin. Cancer Res. Off. J. Am. Assoc. Cancer Res..

[21] Capasso M., Diskin S.J., Totaro F., Longo L., De Mariano M., Russo R., Cimmino F., Hakonarson H., Tonini G.P., Devoto M. (2013). Replication of GWAS-identified neuroblastoma risk loci strengthens the role of BARD1 and affirms the cumulative effect of genetic variations on disease susceptibility. Carcinogenesis.

[22] Ryser S., Dizin E., Jefford C.E., Delaval B., Gagos S., Christodoulidou A., Krause K.H., Birnbaum D., Irminger-Finger I. (2009). Distinct roles of BARD1 isoforms in mitosis: Full-Length BARD1 mediates Aurora B degradation, cancer-associated BARD1β scaffolds Aurora B and BRCA2. Cancer Res..

[23] Pilyugin M., Irminger-Finger I. (2014). Long non-coding RNA and microRNAs might act in regulating the expression of BARD1 mRNAs. Int. J. Biochem. Cell Biol..

[24] Norquist B.M., Harrell M.I., Brady M.F., Walsh T., Lee M.K., Gulsuner S., Bernards S.S., Casadei S., Yi Q., Burger R.A. (2016). Inherited Mutations in Women With Ovarian Carcinoma. JAMA Oncol..

[25] Thai T.H., Du F., Tsan J.T., Jin Y., Phung A., Spillman M.A., Massa H.F., Muller C.Y., Ashfaq R., Mathis J.M. (1998). Mutations in the BRCA1-associated RING domain (BARD1) gene in primary breast, ovarian and uterine cancers. Hum. Mol. Genet..

[26] Stacey S.N., Sulem P., Johannsson O.T., Helgason A., Gudmundsson J., Kostic J.P., Kristjansson K., Jonsdottir T., Sigurdsson H., Hrafnkelsson J. (2006). The BARD1 Cys557Ser variant and breast cancer risk in Iceland. PLoS Med..

[27] Guenard F., Labrie Y., Ouellette G., Beauparlant C.J., Durocher F., BRCAs I. (2009). Genetic sequence variations of *BRCA1*-interacting genes *AURKA, BAP1, BARD1* and *DHX9* in French Canadian families with high risk of breast cancer. J. Hum. Genet..

[28] De Brakeleer S., De Greve J., Loris R., Janin N., Lissens W., Sermijn E., Teugels E. (2010). Cancer predisposing missense and protein truncating BARD1 mutations in non-BRCA1 or BRCA2 breast cancer families. Hum. Mutat..

[29] Couch F.J., Shimelis H., Hu C., Hart S.N., Polley E.C., Na J., Hallberg E., Moore R., Thomas A., Lilyquist J. (2017). Associations between cancer predisposition testing panel genes and breast cancer. JAMA Oncol..

[30] Ratajska M., Antoszewska E., Piskorz A., Brozek I., Borg A., Kusmierek H., Biernat W., Limon J. (2012). Cancer predisposing BARD1 mutations in breast-ovarian cancer families. Breast Cancer Res. Treat..

[31] Ratajska M., Matusiak M., Kuzniacka A., Wasag B., Brozek I., Biernat W., Koczkowska M., Debniak J., Sniadecki M., Kozlowski P. (2015). Cancer predisposing BARD1 mutations affect exon skipping and are associated with overexpression of specific BARD1 isoforms. Oncol. Rep..

[32] Lasorsa V.A., Formicola D., Pignataro P., Cimmino F., Calabrese F.M., Mora J., Esposito M.R., Pantile M., Zanon C., De Mariano M. (2016). Exome and deep sequencing of clinically aggressive neuroblastoma reveal somatic mutations that affect key pathways involved in cancer progression. Oncotarget.

[33] Pugh T.J., Morozova O., Attiyeh E.F., Asgharzadeh S., Wei J.S., Auclair D., Carter S.L., Cibulskis K., Hanna M., Kiezun A. (2013). The genetic landscape of high-risk neuroblastoma. Nat. Genet..

[34] Silversides C.K., Lionel A.C., Costain G., Merico D., Migita O., Liu B., Yuen T., Rickaby J., Thiruvahindrapuram B., Marshall C.R. (2012). Rare copy number variations in adults with tetralogy of Fallot implicate novel risk gene pathways. PLoS Genet..

[35] Faingold R., Babyn P.S., Yoo S.J., Dipchand A.I., Weitzman S. (2003). Neuroblastoma with atypical metastases to cardiac and skeletal muscles: MRI features. Pediatr. Radiol..

[36] George R.E., Lipshultz S.E., Lipsitz S.R., Colan S.D., Diller L. (2004). Association between congenital cardiovascular malformations and neuroblastoma. J. Pediatr..

[37] Caminsky N.G., Mucaki E.J., Perri A.M., Lu R., Knoll J.H., Rogan P.K. (2016). Prioritizing variants in complete hereditary breast and ovarian cancer genes in patients lacking known BRCA mutations. Hum. Mutat..

[38] McDaniel L.D., Conkrite K.L., Chang X., Capasso M., Vaksman Z., Oldridge D.A., Zachariou A., Horn M., Diamond M., Hou C. (2017). Common variants upstream of *MLF1* at 3q25 and within *CPZ* at 4p16 associated with neuroblastoma. PLoS Genet..

[39] Capasso M., McDaniel L.D., Cimmino F., Cirino A., Formicola D., Russell M.R., Raman P., Cole K.A., Diskin S.J. (2017). The functional variant rs34330 of *CDKN1B* is associated with risk of neuroblastoma. J. Cell. Mol. Med..

[40] Oldridge D.A., Wood A.C., Weichert-Leahey N., Crimmins I., Sussman R., Winter C., McDaniel L.D., Diamond M., Hart L.S., Zhu S. (2015). Genetic predisposition to neuroblastoma mediated by a *LMO1* super-enhancer polymorphism. Nature.

[41] Capasso M., Diskin S., Cimmino F., Acierno G., Totaro F., Petrosino G., Pezone L., Diamond M., McDaniel L., Hakonarson H. (2014). Common genetic variants in *NEFL* influence gene expression and neuroblastoma risk. Cancer Res..

[42] Diskin S.J., Capasso M., Diamond M., Oldridge D.A., Conkrite K., Bosse K.R., Russell M.R., Iolascon A., Hakonarson H., Devoto M. (2014). Rare variants in *TP53* and susceptibility to neuroblastoma. J. Natl. Cancer Inst..

[43] Diskin S.J., Capasso M., Schnepp R.W., Cole K.A., Attiyeh E.F., Hou C., Diamond M., Carpenter E.L., Winter C., Lee H. (2012). Common variation at 6q16 within *HACE1* and *LIN28B* influences susceptibility to neuroblastoma. Nat. Genet..

[44] Nguyen le B., Diskin S.J., Capasso M., Wang K., Diamond M.A., Glessner J., Kim C., Attiyeh E.F., Mosse Y.P., Cole K. (2011). Phenotype restricted genome-wide association study using a gene-centric approach identifies three low-risk neuroblastoma susceptibility Loci. PLoS Genet..

[45] Wang K., Diskin S.J., Zhang H., Attiyeh E.F., Winter C., Hou C., Schnepp R.W., Diamond M., Bosse K., Mayes P.A. (2011). Integrative genomics identifies *LMO1* as a neuroblastoma oncogene. Nature.

[46] Capasso M., Devoto M., Hou C., Asgharzadeh S., Glessner J.T., Attiyeh E.F., Mosse Y.P., Kim C., Diskin S.J., Cole K.A. (2009). Common variations in *BARD1* influence susceptibility to high-risk neuroblastoma. Nat. Genet..

[47] Maris J.M., Mosse Y.P., Bradfield J.P., Hou C., Monni S., Scott R.H., Asgharzadeh S., Attiyeh E.F., Diskin S.J., Laudenslager M. (2008). Chromosome 6p22 locus associated with clinically aggressive neuroblastoma. N. Engl. J. Med..

[48] Latorre V., Diskin S.J., Diamond M.A., Zhang H., Hakonarson H., Maris J.M., Devoto M. (2012). Replication of neuroblastoma SNP association at the *BARD1* locus in African-Americans. Cancer Epidemiol. Biomark. Prev..

[49] Zhang R., Zou Y., Zhu J., Zeng X., Yang T., Wang F., He J., Xia H. (2016). The Association between GWAS-identified *BARD1* Gene SNPs and Neuroblastoma Susceptibility in a Southern Chinese Population. Int. J. Med. Sci..

[50] Cimmino F. (2017). BARD1 locus of neuroblastoma susceptibility.

[51] Bosse K.R., Diskin S.J., Cole K.A., Wood A.C., Schnepp R.W., Norris G., Nguyen L.B., Jagannathan J., Laquaglia M., Winter C. (2012). Common variation at *BARD1* results in the expression of an oncogenic isoform that influences neuroblastoma susceptibility and oncogenicity. Cancer Res..

[52] Capasso M., Calabrese F.M., Iolascon A., Mellerup E. (2014). Combinations of genetic data in a study of neuroblastoma risk genotypes. Cancer Genet..

[53] Fu W., Zhu J., Xiong S.W., Jia W., Zhao Z., Zhu S.B., Hu J.H., Wang F.H., Xia H., He J. (2017). *BARD1* gene polymorphisms confer nephroblastoma susceptibility. EBioMedicine.

[54] Gonzalez-Hormazabal P., Reyes J.M., Blanco R., Bravo T., Carrera I., Peralta O., Gomez F., Waugh E., Margarit S., Ibanez G. (2012). The BARD1 Cys557Ser variant and risk of familial breast cancer in a South-American population. Mol. Biol. Rep..

[55] Jakubowska A., Cybulski C., Szymanska A., Huzarski T., Byrski T., Gronwald J., Debniak T., Gorski B., Kowalska E., Narod S.A. (2008). BARD1 and breast cancer in Poland. Breast Cancer Res. Treat..

[56] Spurdle A.B., Marquart L., McGuffog L., Healey S., Sinilnikova O., Wan F., Chen X., Beesley J., Singer C.F., Dressler A.C. (2011). Common genetic variation at *BARD1* is not associated with breast cancer risk in *BRCA1* or *BRCA2* mutation carriers. Cancer Epidemiol. Biomark. Prev..

[57] Ding D.P., Zhang Y., Ma W.L., He X.F., Wang W., Yu H.L., Guo Y.B., Zheng W.L. (2011). Lack of association between BARD1 Cys557Ser variant and breast cancer risk: A meta-analysis of 11,870 cases and 7687 controls. J. Cancer Res. Clin. Oncol..

[58] Johnatty S.E., Beesley J., Chen X., Hopper J.L., Southey M.C., Giles G.G., Goldgar D.E., Chenevix-Trench G., Spurdle A.B. (2009). The *BARD1* Cys557Ser polymorphism and breast cancer risk: An Australian case-control and family analysis. Breast Cancer Res. Treat..

[59] Robinson D.R., Wu Y.M., Lonigro R.J., Vats P., Cobain E., Everett J., Cao X., Rabban E., Kumar-Sinha C., Raymond V. (2017). Integrative clinical genomics of metastatic cancer. Nature.

[60] Carter H., Chen S., Isik L., Tyekucheva S., Velculescu V.E., Kinzler K.W., Vogelstein B., Karchin R. (2009). Cancer-specific high-throughput annotation of somatic mutations: Computational prediction of driver missense mutations. Cancer Res..

[61] Carter H., Douville C., Stenson P.D., Cooper D.N., Karchin R. (2013). Identifying Mendelian disease genes with the variant effect scoring tool. BMC Genom..

[62] Starita L.M., Machida Y., Sankaran S., Elias J.E., Griffin K., Schlegel B.P., Gygi S.P., Parvin J.D. (2004). BRCA1-dependent ubiquitination of gamma-tubulin regulates centrosome number. Mol. Cell. Biol..

[63] Hsu L.C., Doan T.P., White R.L. (2001). Identification of a gamma-tubulin-binding domain in BRCA1. Cancer Res..

[64] Westermark U.K., Reyngold M., Olshen A.B., Baer R., Jasin M., Moynahan M.E. (2003). BARD1 participates with BRCA1 in homology-directed repair of chromosome breaks. Mol. Cell. Biol..

[65] Joukov V., Groen A.C., Prokhorova T., Gerson R., White E., Rodriguez A., Walter J.C., Livingston D.M. (2006). The BRCA1/BARD1 heterodimer modulates ran-dependent mitotic spindle assembly. Cell.

[66] Zhao W., Manley J.L. (1998). Deregulation of poly(A) polymerase interferes with cell growth. Mol. Cell. Biol..

[67] Kleiman F.E., Manley J.L. (2001). The BARD1-CstF-50 interaction links mRNA 3′ end formation to DNA damage and tumor suppression. Cell.

[68] Dechend R., Hirano F., Lehmann K., Heissmeyer V., Ansieau S., Wulczyn F.G., Scheidereit C., Leutz A. (1999). The Bcl-3 oncoprotein acts as a bridging factor between NF-κB/Rel and nuclear co-regulators. Oncogene.

[69] Li M., Yu X. (2013). Function of BRCA1 in the DNA damage response is mediated by ADP-ribosylation. Cancer Cell.

[70] Feki A., Jefford C.E., Berardi P., Wu J.Y., Cartier L., Krause K.H., Irminger-Finger I. (2005). BARD1 induces apoptosis by catalysing phosphorylation of p53 by DNA-damage response kinase. Oncogene.

[71] Irminger-Finger I., Leung W.C., Li J., Dubois-Dauphin M., Harb J., Feki A., Jefford C.E., Soriano J.V., Jaconi M., Montesano R. (2001). Identification of BARD1 as mediator between proapoptotic stress and p53-dependent apoptosis. Mol. Cell.

[72] Jefford C.E., Feki A., Harb J., Krause K.H., Irminger-Finger I. (2004). Nuclear-cytoplasmic translocation of BARD1 is linked to its apoptotic activity. Oncogene.

[73] Choudhury A.D., Xu H., Baer R. (2004). Ubiquitination and proteasomal degradation of the BRCA1 tumor suppressor is regulated during cell cycle progression. J. Biol. Chem..

[74] Hayami R., Sato K., Wu W., Nishikawa T., Hiroi J., Ohtani-Kaneko R., Fukuda M., Ohta T. (2005). Down-regulation of BRCA1-BARD1 ubiquitin ligase by CDK2. Cancer Res..

[75] Smith P.D., Crossland S., Parker G., Osin P., Brooks L., Waller J., Philp E., Crompton M.R., Gusterson B.A., Allday M.J. (1999). Novel p53 mutants selected in BRCA-associated tumours which dissociate transformation suppression from other wild-type p53 functions. Oncogene.

[76] Phillips K.A., Nichol K., Ozcelik H., Knight J., Done S.J., Goodwin P.J., Andrulis I.L. (1999). Frequency of p53 mutations in breast carcinomas from Ashkenazi Jewish carriers of BRCA1 mutations. J. Natl. Cancer Inst..

[77] Li L., Ryser S., Dizin E., Pils D., Krainer M., Jefford C.E., Bertoni F., Zeillinger R., Irminger-Finger I. (2007). Oncogenic BARD1 isoforms expressed in gynecological cancers. Cancer Res..

[78] Zhang Y.Q., Bianco A., Malkinson A.M., Leoni V.P., Frau G., De Rosa N., Andre P.A., Versace R., Boulvain M., Laurent G.J. (2012). BARD1: An independent predictor of survival in non-small cell lung cancer. Int. J. Cancer.

[79] Feki A., Jefford C.E., Durand P., Harb J., Lucas H., Krause K.H., Irminger-Finger I. (2004). BARD1 expression during spermatogenesis is associated with apoptosis and hormonally regulated. Biol. Reprod..

[80] Tsuzuki M., Wu W., Nishikawa H., Hayami R., Oyake D., Yabuki Y., Fukuda M., Ohta T. (2006). A truncated splice variant of human BARD1 that lacks the RING finger and ankyrin repeats. Cancer Lett..

[81] Li L., Cohen M., Wu J., Sow M.H., Nikolic B., Bischof P., Irminger-Finger I. (2007). Identification of BARD1 splice-isoforms involved in human trophoblast invasion. Int. J. Biochem. Cell Biol..

[82] Tembe V., Henderson B.R. (2007). BARD1 translocation to mitochondria correlates with Bax oligomerization, loss of mitochondrial membrane potential and apoptosis. J. Biol. Chem..

[83] Dizin E., Irminger-Finger I. (2010). Negative feedback loop of BRCA1-BARD1 ubiquitin ligase on estrogen receptor α stability and activity antagonized by cancer-associated isoform of BARD1. Int. J. Biochem. Cell Biol..

[84] Pilyugin M., Andre P.A., Ratajska M., Kuzniacka A., Limon J., Tournier B.B., Colas J., Laurent G., Irminger-Finger I. (2017). Antagonizing functions of BARD1 and its alternatively spliced variant BARD1δ in telomere stability. Oncotarget.

[85] Lepore I., Dell’Aversana C., Pilyugin M., Conte M., Nebbioso A., De Bellis F., Tambaro F.P., Izzo T., Garcia-Manero G., Ferrara F. (2013). HDAC inhibitors repress BARD1 isoform expression in acute myeloid leukemia cells via activation of miR-19a and/or b. PLoS ONE.

[86] Capasso M., Diskin S.J. (2010). Genetics and genomics of neuroblastoma. Cancer Treat. Res..

[87] Bryant H.E., Schultz N., Thomas H.D., Parker K.M., Flower D., Lopez E., Kyle S., Meuth M., Curtin N.J., Helleday T. (2005). Specific killing of BRCA2-deficient tumours with inhibitors of poly(ADP-ribose) polymerase. Nature.

[88] Ledermann J., Harter P., Gourley C., Friedlander M., Vergote I., Rustin G., Scott C.L., Meier W., Shapira-Frommer R., Safra T. (2014). Olaparib maintenance therapy in patients with platinum-sensitive relapsed serous ovarian cancer: A preplanned retrospective analysis of outcomes by *BRCA* status in a randomised phase 2 trial. Lancet Oncol..

[89] Lord C.J., Ashworth A. (2013). Mechanisms of resistance to therapies targeting BRCA-mutant cancers. Nat. Med..

[90] Ozden O., Bishehsari F., Bauer J., Park S.H., Jana A., Baik S.H., Sporn J.C., Staudacher J.J., Yazici C., Krett N. (2016). Expression of an oncogenic BARD1 splice variant impairs homologous recombination and predicts response to PARP-1 inhibitor therapy in colon cancer. Sci. Rep..

[91] Pilyugin M., Descloux P., Andre P.A., Laszlo V., Dome B., Hegedus B., Sardy S., Janes S., Bianco A., Laurent G.J. (2017). BARD1 serum autoantibodies for the detection of lung cancer. PLoS ONE.

